# Dendritic Cells in Shaping Anti-Tumor T Cell Response

**DOI:** 10.3390/cancers16122211

**Published:** 2024-06-13

**Authors:** Luciano Mazzoccoli, Bei Liu

**Affiliations:** 1 Division of Hematology, Department of Internal Medicine, The Ohio State University Comprehensive Cancer Center, Columbus, OH 43210, USA; luciano.mazzoccoli@osumc.edu; 2 The Pelotonia Institute for Immuno-Oncology, The Ohio State University Comprehensive Cancer Center, Columbus, OH 43210, USA

**Keywords:** dendritic cells, antigen presentation, tumor-infiltrating T cells, tumor microenvironment, cancer

## Abstract

**Simple Summary:**

Cancer stands as the second leading cause of death globally. Over the years, concerted efforts have been devoted to developing innovative and effective therapies for tumor eradication and to prevent recurrence. A critical factor contributing to therapy failures is the patient’s immune response. Consequently, recent endeavors, such as immune checkpoint blockade, adoptive cell therapy, monoclonal antibodies, and cancer vaccines, aim to enhance a patient’s immune system, maximizing the benefits of therapies for tumor clearance and subsequent memory formation. Dendritic cells (DCs) play a pivotal role in regulating the immune response. DCs are a heterogeneous population with varying functions in the tumor microenvironment. Reprogramming tumor-infiltrating DCs can dramatically improve the immune response against tumors and has shown significant therapeutic potential in pre-clinical models. In this review, we summarize the subsets and functions of dendritic cells and their role in shaping the anti-tumor T cell immune response.

**Abstract:**

Among professional antigen-presenting cells, dendritic cells (DCs) orchestrate innate and adaptive immunity and play a pivotal role in anti-tumor immunity. DCs are a heterogeneous population with varying functions in the tumor microenvironment (TME). Tumor-associated DCs differentiate developmentally and functionally into three main subsets: conventional DCs (cDCs), plasmacytoid DCs (pDCs), and monocyte-derived DCs (MoDCs). There are two major subsets of cDCs in TME, cDC1 and cDC2. cDC1 is critical for cross-presenting tumor antigens to activate cytotoxic CD8^+^ T cells and is also required for priming earlier CD4^+^ T cells in certain solid tumors. cDC2 is vital for priming anti-tumor CD4^+^ T cells in multiple tumor models. pDC is a unique subset of DCs and produces type I IFN through TLR7 and TLR9. Studies have shown that pDCs are related to immunosuppression in the TME through the secretion of immunosuppressive cytokines and by promoting regulatory T cells. MoDCs differentiate separately from monocytes in response to inflammatory cues and infection. Also, MoDCs can cross-prime CD8^+^ T cells. In this review, we summarize the subsets and functions of DCs. We also discuss the role of different DC subsets in shaping T cell immunity in TME and targeting DCs for potential immunotherapeutic benefits against cancer.

## 1. Introduction

Among professional antigen-presenting cells (APCs), dendritic cells (DCs) orchestrate both innate and adaptive immunity [1] and are responsible for the balance between immunity and tolerance [2]. Although the role of APCs in activating T cells has been known since the 1960s [3], DCs were first described by Ralph Steinman and Zanvil Cohn as initiators of T cell priming in 1973 [4]. As important players in immune surveillance, DCs are constantly capturing dead cells through endocytosis and processing the proteins to further present them to naïve T cells in the peripheral lymphoid organs where antigen presentation and T cell activation occur. As for cell activation, it has been recently shown that DCs are also able to present antigens to B cells [5], although further investigations to clarify the process are needed.

DCs are a heterogeneous population with varying functions in the tumor microenvironment (TME) [6]. Tumor-associated DCs differentiate developmentally and functionally into three main populations: conventional DCs (cDCs), plasmacytoid DCs (pDCs), and monocyte-derived inflammatory DCs (infDCs) [7,8]. Intratumoral cDCs are capable of maturing into inflammatory or regulatory DCs. Two major cDC subsets exist within tumors, type 1 cDC (cDC1) and type 2 cDC (cDC2). cDC1 is differentiated by expression of CD103/XCR1/Clec9A in mice and CD141/XCR1/Clec9A in humans, while cDC2 is classified by expression of CD11b/CD172 in mice and CD1c in humans [9]. cDC1 is critical for cross-presenting tumor antigens to activate CD8^+^ T cells [6]. A recent study showed that cDC1s are also required for earlier CD4^+^ T cell priming, and CD40 signaling in cDC1 is critical for CD8^+^ T cell priming and CD4^+^ T cell activation against fibrosarcoma and melanoma [10]. The literature reports that intratumoral cDC2 could migrate to tumor-draining lymph nodes (dLN) and present tumor antigens to CD4^+^ T cells. cDC2 is required for improved CD4^+^ T cell priming and tumor rejection in the absence of regulatory T cells (Tregs) [11]. Interestingly, a more recent study demonstrated that an activation state of tumor-infiltrating CD11b^+^ cDC2s expressing an IFN-stimulated gene (ISG) signature presented a tumor-derived peptide-MHC I complex to activate CD8^+^ T cells and promoted anti-tumor immunity in the absence of cDC1s [12]. However, cDC1 also plays an immunosuppressive function by secreting anti-inflammatory cytokines or by expressing checkpoint molecules, which inhibit T cell activation. Studies showed that intratumoral CD103^+^ cDC1s highly expressed TIM-3 in breast cancer, targeting a TIM-3 improved response to chemotherapy and enhanced CD8^+^ T cell activation via cDC1-derived cytokines [13,14]. Additionally, during tumor progression, immature cDCs can be converted to regulatory DCs, which play a major role in inhibiting anti-tumor immune responses through the induction of regulatory T cells [15]. These regulatory DCs have been reported in numerous cancers, including ovarian, breast, colon, and lung [16,17,18,19,20]. Reprogramming tumor-infiltrating DCs (TiDCs) can dramatically improve the immune response against tumors and has shown significant therapeutic potential in pre-clinical models [18,19,21,22,23,24]. In this review, we summarize the subsets and functions of DCs. We also discuss the roles of different DC subsets in shaping T cell immunity in the TME and targeting DCs for potential immunotherapeutic benefit against cancer.

## 2. Subsets of Dendritic Cells

**Figure 1:**
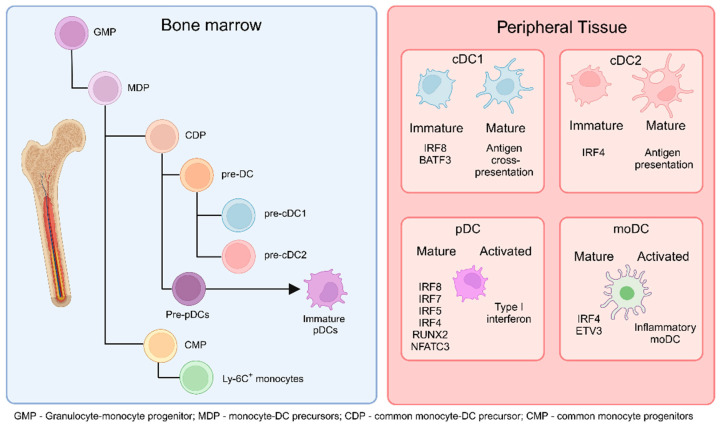
DCs originate from common monocyte-DC precursors (MDP) in the bone marrow (BM), which arise from granulocyte-monocyte progenitors (GMP). MDPs further differentiate into common DC precursors (CDP) or common monocyte progenitors (CMP). CDPs subsequently give rise to pre-cDC1 and pre-cDC2 cells. Additionally, CDPs can differentiate into pre-plasmacytoid DCs (pre-pDCs), which further mature into immature pDCs. CMPs, on the other hand, differentiate into monocytes. The cells generated in the BM migrate to peripheral tissues and secondary lymphoid organs as immature or not fully mature cells. Upon encountering activation conditions, immature cDC1 cells fully mature to cross-present antigens to T cells in the lymphoid organs, cDC2 cells present antigens and produce cytokines, pDCs increase type I interferon production, and monocyte-derived DCs (moDCs) differentiate into inflammatory moDCs. (Created via BioRender).

**Figure 2:**
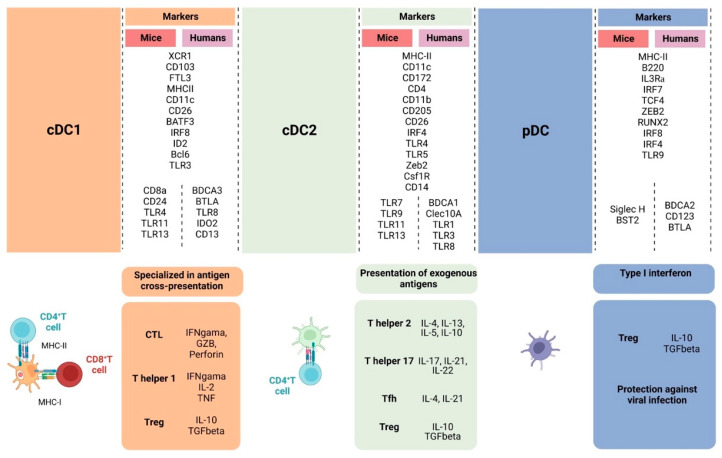
Overall illustration of cell-surface markers and transcriptional factors shared between humans and mice and singularities of cDC1, cDC2, and pDC. cDC1 is specialized in activating naïve CD8^+^ T cells through the cross-presentation of antigens, inducing CTL against virally infected cells and tumor cells and Th1 differentiation, which will support the anti-tumor response through IFN-γ, IL-2, and TNF-α production. cDC2 induces CD4^+^ T cells to differentiate into different types of T helper cells that have distinct roles in the anti-tumor response, supporting CTL differentiation and effector functions. Depending on the activation status and TME, DCs can also contribute to Treg cell development. pDCs are effective against viral infection through type I interferon production but also can lead to Treg expansion through indoleamine 2,3-dioxygenase and PD-L1 expression. (Created via BioRender).

DCs are a heterogeneous population with varying functions. Tumor-associated DCs can be differentiated developmentally and functionally into three main populations: cDCs, pDCs, and infDCs [7,8], which are differentiated from different progenitors in the bone marrow (Figure 1) and are characterized by different transcription factors and cell-surface markers in mice and humans (Figure 2).

### 2.1. Conventional Dendritic Cells

Two major cDC subsets exist within tumors, known as cDC1 and cDC2. These cDC subsets are distinguished by their expression of different transcription factors. cDC1 is primarily defined by the transcription factors basic leucine zipper transcription factor (BATF3) and interferon regulatory factor 8 (IRF8). The development of cDC1 requires the transcription factors IRF8 and BATF3, essential for pre-cDC1 committed to cDC1 [25]. The genesis of DC occurs in the bone marrow to further pre-cDC in both mice and humans. Also, the cytokine FLT3L is essential for cDC development in both mice and humans [26]. cDC1s are also defined based on the cell-surface expression of CD8α and CD103, with CD8α^+^ cDC1s referring to mice, as humans do not express CD8α. In humans, cDC1 is characterized by HLA-DR^high^, CD11c^int^, and CD141^high^, and the lack of lineage (Lin) markers CD19, CD14, CD16, and CD3. Other common shared markers between mice and humans are CADM1, Clec9a, and XCR1 [27,28]. In mice, the chemokine receptor XCR1 is essential to characterize cDC1. Also, murine cDC1 lacks the expression of macrophage markers F4/80, CD64, Cd11b, and CD172 [9,29] (Figure 2).

cDC2 is characterized by MHC II^high^, CD11c^high^, CD11b^high^, and CD172^+^ in mice, and by HLA-DR^+^, CLEC10A^+^, CD1c^+^, and CD172^+^ in humans [29,30]. In mice, cDC2 expresses the migratory marker CD103^+^ in intestinal tissue [26]. As for unique transcription factors, cDC2 depends on interferon regulatory factor 4 (IRF4), zinc finger E-box-binding homeobox 2 (ZEB2), and NOTCH2/KLF4 [29,31]. In conditional knockout for IRF4, cDC2 is still present in the skin, but the decreased frequency in other tissues denotes a different dependency on IRF4 for development, survival, and migration [32,33]. Using single-cell RNA-sequencing (scRNA-seq), ATAC-sequencing, and specific gene reporter analyses, the researchers discovered two cDC2 subtypes, cDC2A (T-bet^+^) and cDC2B (T-bet^-^), emphasizing the heterogeneity of cDC2s across mouse and human [34,35,36]. Functionally, cDC2 activates CD4^+^ T cells and supports the CD8^+^ T cell immune response, allowing CD4^+^ T cells to polarize into different T helpers (Th2, Th17, Treg). A recent study has shown that in BATF3-deficient mice, type I interferon (IFN) induced stimulatory cDC2 MHC-I-dressed to activate CD8^+^ T cells, a process that was enhanced by IFN-β [12] (Figure 2).

Using scRNA-seq and CITE-sequencing approaches, recent studies identified a cluster of mature DC enriched in regulatory molecules (mregDC) in the lung TME. cDC1 and cDC2 can differentiate into mregDCs upon tumor-antigen uptake that limits anti-tumor responses in human and mouse cancers [37,38,39,40,41]. A more recent study showed that mregDCs existed in head and neck squamous cell carcinoma (HNSCC) TME and had migratory and mature phenotypes. The mregDC signature genes were strongly associated with Treg signature genes in HNSCC [42]. The mregDCs present a regulatory activity toward tumor-responding T cells. The mregDCs are defined by the coexistence of a maturation profile (such as CD80, CD86, and LAMP3), a migration profile (such as CCR7), and an immunoregulatory profile (such as PD-L1/PD-L2, IDO1, and TIM-3) [37,40,41,42,43]. The mregDCs play a critical role in balancing Tregs and effector T cells in the TME.

### 2.2. Plasmacytoid Dendritic Cells

pDCs are a unique subset of DCs and differ from cDCs in morphology, resembling antibody-secreting plasma cells. The origin of pDCs is from myeloid and lymphoid. pDCs are reported to express MHC class II (MHC II), preserving the function of APC and mostly secreting type I IFN in responses involving viral infection and anti-tumor responses in humans and mice [44,45]. Human pDCs are defined by expressing BDCA2 (CD303), BDCA4 (CD304), and IL-3 receptor (CD123), and not expressing Lin markers CD3, CD11c, CD14, CD16, and CD19. In mice, pDCs are characterized by expressing a high level of MHC II and intermediate levels of CD11c and B220, but also by a lack of Lin markers [46,47]. These markers with sialic acid binding Ig-like lectin H (Siglec-H) are used to distinguish pDCs from other DCs [48]. IRF8 is also involved in pDC differentiation along with RUNX1 and TCF4 [49]. Upon maturation, downregulation of Siglec-H has been reported. Tumor-infiltrating pDCs have both negative and positive impacts depending on the tumor type [50]. pDCs can present antigens and activate CD8^+^ T cells via MHCII and co-stimulating molecules CD40, CD80, and CD86. Depending on the context, pDCs can also act as tolerogenic through the expression of immunosuppressive molecules indoleamine 2,3-dioxygenase and PD-L1, and by favoring Treg expansion [46,51] (Figure 2).

### 2.3. Monocyte-Derived Dendritic Cells

Monocyte-derived DCs (MoDCs) are differentiated from monocytes and recruited to tissues in response to inflammatory cues and infection [52,53]. Inflammatory MoDCs can program CD4^+^ T cells to Th1, Th2, and Th17 phenotypes [54]. MoDCs also can cross-present antigens to prime the CD8^+^ T cell response. However, the transcriptional program in MoDCs is distinct from cDCs. GM-CSF-derived MoDCs depend on IRF4 and IL-4 for cross-presentation but not Batf3 [55]. MoDCs share some phenotypical and functional features with monocytes and cDC2. A recent study demonstrated that two subtypes of MoDCs (MoDC1 and MoDC2) differentiated from Ly6C^hi^ monocytes in mouse TME. MoDCs expressed high levels of MHC I, MHCII, and CD86. MoDC1 expressed PD-L1 while MoDC2 mostly expressed CD155 [56]. In human melanoma, MoDCs expressed high levels of HLA-DR, CD1c, CLEC10A, CD11c, and cystatin F (CST7). Also, CD86, PU.1, ILT3, CSF1R, and CD11b were upregulated in MoDCs and cDC2 in PD1 checkpoint blockade responders [56]. Since MoDCs overlap with other DC subsets, further studies are needed to identify MoDCs specifically.

## 3. Functions of Dendritic Cells

### 3.1. Antigen Uptake

DCs internalize antigens through various mechanisms, including phagocytosis, micro- or macro-pinocytosis, and endocytosis. This process involves membrane receptors such as antibody receptor type I or II (Fcγ I or Fcγ II), receptors involved in cell attachment to the extracellular matrix (integrins), glycan-binding proteins involved in cell-to-cell interaction (lectin), receptors for apoptosis signals through phosphatidylserine (PS), phagocytic receptors, and scavenger receptors. Subsequently, antigens undergo processing either via the endogenous pathway, leading to their presentation on MHC class I molecules to CD8^+^ T cells, or via the exogenous pathway, resulting in their presentation on MHC class II molecules to CD4^+^ T cells [26,57,58,59,60]. A previous study showed that cDC1 from the lung expresses TIM4, a phosphatidylserine receptor that mediates the engulfment of cell-associated antigens and the activation of several processes [61].

**Figure 3:**
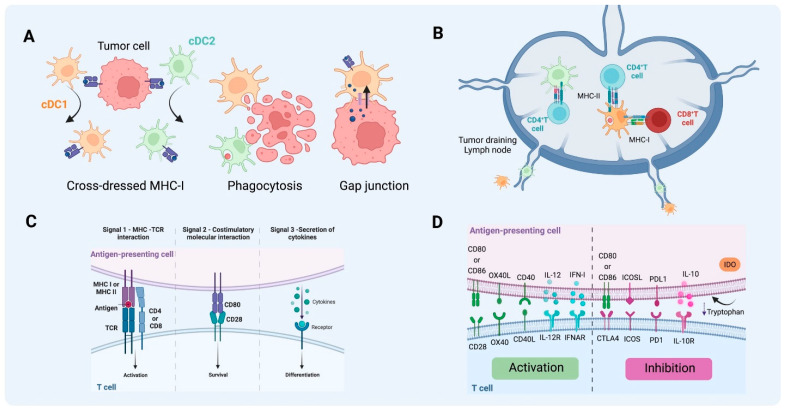
Antigen uptake and presentation by DCs in T cell activation. (**A**) Different pathways of antigen acquisition involving phagocytosis of apoptotic bodies or immunogenic cell death. Instead of capturing tumor proteins and further processing them into peptides to present through MHC-I, cDC1 and cDC2 can also obtain fragments of tumor membrane, a process referred to as cross-dressing through trogocytosis. In this process, MHC-I loaded with peptides from tumor cells can be harvested and further used for presentation to T cells. Another described way to obtain peptides already processed from non-antigen-presenting cells is through gap junctions between APC and tumor cells. (**B**) T cell activation by DCs on tumor-draining lymph nodes. cDC1 can present antigen to CD4^+^ T cells through MHC II and cross-present antigen to naïve CD8^+^ T cells through MHC-I. As for cDC2, the acquisition of exogenous antigens can lead to MHC-II antigen presentation to naïve CD4^+^ T cells that will further differentiate into different types of T helper, depending on the context and cytokine milieu. (**C**) The activation of T cells requires three signals, MHC-antigen interaction, co-stimulatory molecule interaction between T cells and mature DCs, and the third signal commonly delivered by the cytokine milieu. (**D**) Together this will lead to activation or inhibition, depending on the co-stimulatory molecules, cytokines, and byproducts from the metabolism of the cells and TME involved in the process (e.g., tryptophan catabolites released into the TME following the activity of indoleamine 2,3-dioxygenase). (Created via BioRender).

A fundamental mechanism by which DCs capture antigens is phagocytosis. This process resembles a zipper-like action and is actin-driven and receptor-mediated. It entails the participation of membrane phospholipids, small GTPases, kinases, cytoskeletal elements, channels, and numerous proteins working in concert to enable the engulfment of sizable particles (ranging from larger than 0.5 µm up to 10 or even 20 µm in diameter) [62], including microorganisms, foreign substances, and apoptotic cells. Upon antigen uptake, DCs undergo maturation, increasing the expression of MHC II, CD40, CD80, and CD86, the production of cytokines such as IL-12 by cDC1 and other pro-inflammatory cytokines, and the expression of CCR7 as a homing receptor to draining lymph nodes (dLN). Lymphoid tissue such as the spleen and lymph nodes (LN) is critical for sampling blood- and lymph-born antigens, respectively, and DCs can directly transport antigens from the periphery [26]. In murine models, migratory CD103^+^ cDC1s are necessary for transporting cellular antigens from the periphery to the LN [63]. In the TME, CD103^+^ cDC1s actively transport tumor antigens to tumor dLN [64] (Figure 3).

### 3.2. Antigen Processing and Presentation

Depending on the source of antigens, exogenous or endogenous sources, two distinct pathways have been known for the processing and presentation of peptides on MHC molecules. The molecular mechanisms that regulate classical antigen presentation on MHC II molecules and cross-presentation are diverse. Two major pathways are involved in antigen presentation based on which DCs present antigens to activate the adaptive immune response. The direct pathway involves MHC-II molecules and CD4^+^ T cell activation, and the source of antigens comes from the extracellular environment. The endogenous pathway involves MHC-I molecules and CD8^+^ T cell activation. However, the antigens obtained (exogenous) by DCs that were not infected directly with activation of CD8^+^ T cells to become cytotoxic were denominated as an antigen cross-presentation [65,66].

Cross-presentation encompasses two distinct pathways: the vacuolar pathway and the endosome-to-cytosol pathway. In the vacuolar pathway, antigen processing and the subsequent loading onto MHC I molecules occur within the endo/lysosomal compartment. After internalization, lysosomal proteases break down antigens into peptides, typically eight or nine amino acids. These peptides are then transported into the lumen, where they bind to MHC class I molecules. Conversely, in the endosome-to-cytosol pathway, internalized antigens must traverse from the endosomal compartment into the cytosol for degradation by the proteasome [66]. Following antigen degradation and peptide generation, a transporter associated with antigen processing (TAP) facilitates peptide transportation into the lumen of the endoplasmic reticulum (ER) or antigen-containing endosomes, where they further associate with MHC-I molecules [67].

Internalized antigens are degraded in endo/lysosomal compartments by proteases such as cathepsins and further loaded for MHC II-restricted presentation. Newly synthesized MHC II molecules are stabilized by binding to the invariant chain (Ii) to be further transported from ER to the endosome, where Ii is degraded by lysosomal proteases, allowing the binding of only a peptide fragment (CLIP) to MHC II, and antigen-derived peptides take the place of CLIP by the chaperone HLA-DM [68]. The mature DCs mainly express MHC II on the cell surface. However, immature DCs retain MHC II in late-endosome and lysosomal vesicles [69]. The transportation of material between cytosol and ER with the lumen of endocytic compartments only happens in specific cells. This process allows exogenous antigens to be processed into peptides and further loaded onto MHC I molecules [70].

The antigen solubility can also influence the type of endocytosis and its immunogenicity. Soluble antigens are typically internalized via pinocytosis or receptor-mediated endocytosis, while particulate antigens are captured through phagocytosis [71,72]. Additionally, particulate antigens generally exhibit higher immunogenicity than soluble antigens, which has implications for vaccine-based therapies [73,74]. Soluble antigens are rapidly directed to lysosomal proteases for efficient degradation, resulting in poor cross-presentation. However, when targeted to early endosomes instead of late endosomes that fuse with lysosomes, antigens are shielded from complete degradation, thereby contributing to efficient cross-presentation [75]. Furthermore, antigens encapsulated in large vesicles (~500 nm) are predominantly found in early endosomes, whereas those prepared as smaller particles (<200 nm) are rapidly trafficked to late endosomes with kinetics similar to soluble antigens [76], emphasizing the importance of endosomal antigen stability for efficient antigen cross-presentation.

Additionally, cross-dressing is another mechanism for antigen presentation. DCs can capture exogenous pre-formed peptide-MHC-I complexes (p/MHC) acquired from neighboring cells that often are not APCs, meaning they could also be tumor cells if MHC-I expression is not suppressed [77,78]. DCs present antigens via MHC cross-dressing by two mechanisms. The first is through cell–cell contact-dependent plasma membrane transfer, a process called trogocytosis. The second mechanism is mediated by the secretion of membrane vesicles called exosomes [78]. Recent studies showed that cross-dressing MHC-I by CD103^+^ DCs in vivo was sufficient for priming antigen-specific CD8^+^ T cells, and the impact may differ between tumor models. Also, CD11b^+^ cDC2s expressing an IFN-stimulated gene (ISG) signature presented tumor-derived peptide-MHC I complex to activate CD8^+^ T cells and promoted anti-tumor immunity in the absence of cDC1s [12,77]. Further study is needed to understand how different DC subsets prime T cells through MHC cross-dressing in the TME.

## 4. Dendritic Cells Shape T Cell Response in the TME

Throughout the years, studies have found that the infiltrating immune cells in TME are associated with the prognosis of ovarian, renal cell, colorectal, and breast cancers, with the predominance of CD4^+^ and CD8^+^ T cells, cDCs, macrophages, and Tregs [79,80,81]. Several studies have shown that T cell infiltration predicts a better outcome [79]. From advanced ovarian cancer patient samples, tumor-infiltrating lymphocytes (TILs) were observed in 55% of tumors in patients with a 5-year overall survival rate of 38% in comparison with a 4.5% rate in patients with no TIL. However, tumor-infiltrating FoxP3^+^ Treg cells were associated with poor prognosis [82,83,84].

**Figure 4:**
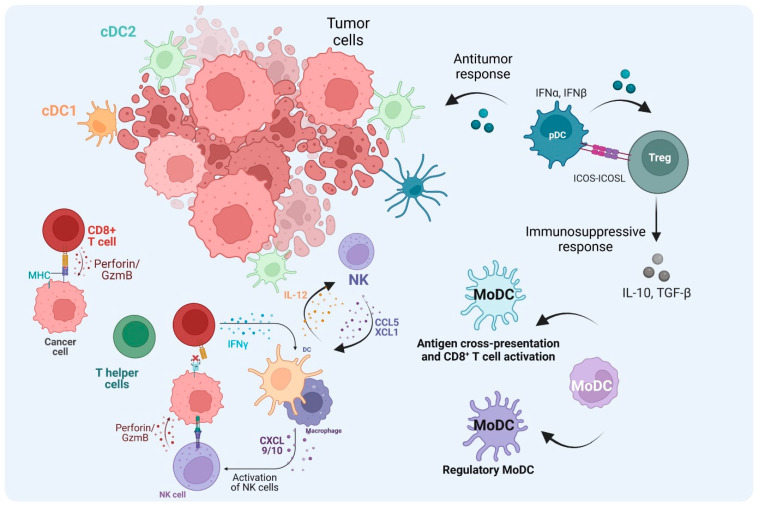
The role of DCs in the TME. After cDC1s uptake and process tumor antigens and become mature cDC1s, they then migrate to lymph nodes and activate naïve CD8^+^ T cells. The activated T cells migrate to the tumor site as CTLs through TCR–MHC-I interaction. CTLs kill tumor cells mediated by perforin and Granzyme B. In the absence of MHC-I from tumor cells, NK cells can mediate the cytotoxic kill, and cDC1 also contributes to the recruitment of NK cells to the TME. Also, NK cells can increase cDC1 influx through CCL5 and XCL1. cDC2s not only activate T helper (Th) cells to promote the anti-tumor immune response through cytokine production but they can also abrogate the effector functions when the Th cells become Treg cells. TME cues, tolerogenic pDCs, and regulatory monocyte-derived DCs (MoDCs) can induce Treg cells, which regulate the immune response contributing to tumor escape. In addition, MoDCs can prime CD8^+^ T cell activation in the TME. (Created via BioRender).

Considering the specialized functions of DCs and their heterogeneity in humans and mice, different subsets of DCs can impact the outcomes in the different TMEs [85,86]. As previously mentioned, cDC1s cross-present antigens to naïve CD8^+^ T cells and activate CD8^+^ T cells to become cytotoxic T cells (Figure 4). Batf3-deficient mice failed to reject melanoma tumors, with less accumulation of CD103^+^ DCs and a decreased CXCL10 in TME, abrogating the recruitment of adoptive T cells to the tumors [87]. Although studies showed a positive correlation between cDC1 and therapeutic response and patient overall survival [31], the role of cDC1 in TME might play different functions depending on the tumor type, as other studies showed contradictory results. Our study showed that cDC2 infiltration significantly correlated with survival across multiple human cancers, with a benefit seen in tumors resistant to cytotoxic T cell control [88]. Also, in Batf3-deficient mice, cDC2s were increased in the dLN and there was improved survival in a breast cancer model [88].

In the TME, CD8+ T cells exhibit a reduced ability to generate pro-inflammatory cytokines such as IFN-γ, TNF-α, and IL-2 and their capacity to kill cancer cells, known as T cell exhaustion. Exhausted T cells increase the expression of several co-inhibitory receptors, including PD-1, TIM3, LAG3, and 2B4 [89]. cDC1 can sustain potentially exhausted T cells (Tpex) in marginal and follicular zones of LN and preserve Tpex to become terminal exhaustion in a chronic infection model [90]. cDC1 has been reported to initially prime naïve CD8^+^ T cells in the tumor-draining LN (dLN) and further on to fully activate the CD8^+^ effector T cells through CD80/CD86, where the gain of cytotoxic capacity requires co-stimulation in the TME [91]. cDC1s also play immunosuppressive functions by secreting anti-inflammatory cytokines or expressing checkpoint molecules to inhibit T cell activation. A recent study showed that intratumoral CD103^+^ cDC1 highly expressed TIM-3 in breast cancer, and that targeting TIM-3 improved the response to chemotherapy [13].

cDC2 can migrate to the dLN and present tumoral antigens to CD4^+^ T cells. During the process, cDC2 becomes fully functional and activates CD4^+^ T cells, which in turn gives rise to Th2 effector cells secreting IL-2 and IFN-γ, contributing to CD8^+^ T cell activation by providing the 3rd signal. cDC2 also enhances the phagocytosis by macrophages and the activation of NK cells, denoting the crucial role of CD4^+^ T cells in the anti-tumor response [92] (Figure 4). Although the ability of cDC2s to cross-present antigens in humans has been reported [93,94,95], it is essential to consider the model used, the efficacy of CTL activity, and the methodology employed to characterize classic cDC2s. Recent innovative approaches in scRNA-seq and spectral flow cytometry have started to shed light on studying the role of cDC2s in the anti-tumor immune response, despite the challenge of distinguishing them from moDCs. Within tumors, cDC2s play a complex role in activating CD4^+^ T cells. Unlike the well-studied CD8^+^ T cells, the function of CD4^+^ T cells in cancer progression and their significance in immunotherapy are not fully understood. Previous studies have highlighted the importance of cDC2s in activating conventional CD4^+^ T cells (Tconv). In the dLN, cDC2s initiate the activation of CD4^+^ Tconv, but their differentiation into effective effector cells is impaired, suggesting that enhancing the priming of CD4^+^ Tconv could be achieved by reprogramming cDC2s. Full CD4^+^ Tconv activation was achieved when Treg cells were depleted. Furthermore, the frequency of cDC2s and Tregs predicted the quality of CD4^+^ Tconv priming observed in both humans and mice [11], potentially predicting the success of immune checkpoint blockade (ICB) therapy. Consistent with shaping immune responses, a recent study demonstrated that CD1c^+^ CD14^+^ DC3-like cells in humans are related to cDC2s rather than moDCs. Under conditions of tumor-derived IL-6 and M-CSF, cDC2s can be converted into DC3-like cells with protumor activity through the expression of IDO, IL-10, PD-L1, and MERTK [96]. DC type 3 (DC3) is another subtype previously mentioned in humans that resembles cDC2 [35,97]. In the presence of IFN-I, cDC2 and DC3 respond differently; DC3 increases glucocorticoid-induced tumor necrosis factor receptor ligand (GITRL), which is suggested to function as signal 4 to provide post-priming signals to T cells and increase the survival of resident memory T cells (T_RM_) [98,99]. Additionally, recent studies showed that DC3 could license CD8^+^ T cells in vivo and increase CD103^+^ T_RM_ infiltration [39,100]. In melanoma patients, it was shown that the presence of DC3 improved overall survival, where tumor samples expressed increased levels of co-stimulatory molecules CD80 and CD86 in DC3 [101]. In a murine model, a study demonstrated a lineage of DC3 that encompasses CD16/32 and CD172α and arises from MDP, differing from cDC2, which arises from CDP [102]. Nonetheless, the ontogeny of DC3 is still not fully understood, and the tumoral milieu can reshape these cells by modulating their transcriptional programs. For example, in organotypic human skin melanoma cultures, immunostimulatory cDC2s were shaped into CD14^+^ DCs with impaired T-cell stimulation capacity [103]. Also, 3D DC-tumor organoids of colorectal cancer cells in vitro showed similar results regarding cDC2 becoming DC3-like and the influence of PGE2 and IL-6 [104]. Contrary to their protumor role, cDC2s are also associated with anti-tumor responses. In non-small-cell lung cancer patients, scRNA-seq analysis to map tumor-infiltrating myeloid cells revealed that among myeloid cells, cDC2s were positively associated with survival [40]. Additionally, our group demonstrated that human breast cancer with a cDC2 signature was strongly correlated with a higher survival rate than cDC1, and in a mouse model, cDC2s contributed to the infiltration of CTLs and M1 macrophages in TME [88]. The role of cDC2s in anti-tumor responses may also depend on the intact presentation of tumor-derived peptide-MHC I complexes [12]. In Batf3^−/−^ mice lacking cDC1, CD11b+ cDC2s expressing interferon (IFN)-stimulated genes (ISGs) (ISG^+^ DCs) activated CD8^+^ T cells ex vivo comparably to cDC1. Unlike cross-presenting cDC1s, ISG^+^ DCs acquired peptide-MHC I complexes from tumor cells, and along with type I IFN and co-stimulatory molecules, ISG^+^ DCs contributed to anti-tumor immunity in the absence of mature cDC1 [12]. Overall, cDC2 may play a critical role when cDC1 is lacking or under the control of the TME, suggesting that cDC2 can also be a potential component in anti-tumor therapeutic strategies.

mregDCs represent a matured state of both conventional dendritic cell subsets, cDC1 and cDC2, unlike DC3, whose progenitor population is distinct from those giving rise to cDC1s and cDC2s [39]. However, it is noteworthy that mregDCs derived from cDC1s and cDC2s exhibit distinct characteristics [105]. Additionally, a comprehensive pan-cancer analysis has elucidated that cDC1-like mregDCs are more predominant in most tumors, whereas cDC2-like mregDCs tend to be more prevalent in pancreatic and nasopharyngeal cancers [106,107]. Within the TME, DC functionality can be regulated by cytokines, with IFNγ promoting IL-12 production and IL-4 suppressing it [37]. Also, blocking IL-4 in tumor-bearing mice led to the restoration of IL-12 production by mregDCs, consequently enhancing anti-tumor immunity [37]. In addition, analysis of hepatocellular carcinoma tumor samples and esophageal cancer revealed that mregDCs can regulate exhausted CD8^+^ T cells and Tregs via PD-1/PDL-1 [42]. Tumor-infiltrating mregDCs abrogate T cell proliferation and effector functions through PD-1/PDL1 interactions when co-cultured with naïve CD8+ T cells [108]. Regarding regulatory molecules on DCs, a recent study revealed that TIM-3 plays a crucial role in mregDC maturation. Furthermore, the Bat3 adapter protein, which binds to the cytoplasmic tail of Tim-3, contributes to DC-mediated tolerance [43,109]. From a genetic knockout mouse model and single-cell RNAseq analysis, previous studies demonstrated that the deletion of TIM-3 on DCs prevented the acquisition of the mregDC program, which plays a pivotal role in preserving stem-like T cells and CD8^+^ effector cells [43]. Additionally, the combined treatment of anti-TIM-3 and anti-PD-L1 significantly reduced tumor burden [43,110]. Furthermore, inhibition of TIM-3 induced cDC1-like mregDCs to uptake tumor antigen and activate the Stimulator of Interferon Genes (STING) pathway, which leads to the release of CXCL9 and IL-12 from mregDCs [43]. It promoted the colocalization of CD8^+^ T cells with DCs, thereby enhancing anti-tumor immunity [109].

pDCs play a critical role in antiviral responses and they secrete type I interferons (IFN-I) during viral infections. Also, pDCs play a pivotal role in anti-tumor immune responses through IFN-I production [100]. However, the role of pDCs within the TME varies depending on the tumor type and cellular interactions, which consequently impact the prognosis. In the context of melanoma, reduced levels of circulating pDCs were associated with a negative impact on overall survival and progression-free survival, and this association was independent of the frequency of myeloid-derived suppressor cells (MDSCs) [111]. The presence of tumor-infiltrating pDCs was correlated with a poor prognosis and was independent of neutrophil infiltration [112]. Both human samples and mouse models have shown that pDCs accumulate at the tumor site, promoting the expression of Th2 inflammatory cytokines such as IL-13 and IL-5, as well as IL-10-secreting Treg cells, which correlated with the expression of OX40L and ICOSL from pDCs [113]. Although a high level of pDCs was associated with late stages in NSCLC [114], another study reported that elevated levels of pDCs were associated with a favorable prognosis and correlated with an enhanced circulating tumor-specific T cell response in NSCLC, despite the absence of direct interactions between pDCs and T cells in the study. Also, patients with a high pDC signature in the TME exhibited a better response to anti-PD-L1 treatment, suggesting that pDC levels could predict responses to ICB therapy [115]. Furthermore, a recent study reported that a high pDC level was correlated with a better prognosis in triple-negative breast cancer (TNBC) but not in other types of breast cancer. Also, tumor-infiltrating pDCs were associated with CD8^+^ and CD4^+^ memory T cells, IFN-γ score, and cytolytic activity in TNBC. Interestingly, the high level of pDCs was correlated with the upregulation of immune checkpoint molecules, including PD-1, PD-L1, PD-L2, CTLA-4, LAG3, and TIGIT, suggesting that patients with high pDC TNBC might respond to ICBs [50]. However, another study showed that GM-CSF skewed pDCs to promote a regulatory Th2 response [116]. Altogether, targeting pDCs may enhance anti-tumor T cell responses in the context of ICB therapy.

MoDCs are another critical player in anti-tumor immunity. MoDC overlaps with cDC2 subsets and monocytes. Programming monocytes into MoDCs plays a role in inflammation and infection [117]. In the presence of IL-4 and IRF4, MoDCs can cross-prime antigens to CD8^+^ T cells [55] (Figure 4). Also, MoDCs express high levels of co-stimulatory molecules in correlation with effector TILs in the TME after ICB treatment. MoDCs and TILs were significantly higher in ICB responders. Intriguingly, targeting MoDCs with agonistic anti-CD40 antibody enhanced PD-1 ICB efficacy, and MoDCs differentiated into iNOS-producing cells to support tumor-infiltrating T cell expansion [56]. Targeting MoDCs may provide insight into developing combination therapies for improving poor ICB response.

The TME’s ability to hinder anti-tumor immunity by establishing an immunotolerant milieu poses a significant obstacle to cancer immunotherapy. To fully leverage the immunogenic potential of DCs in immunotherapy, it is imperative to overcome these immunosuppressive mechanisms.

## 5. Conclusions and Perspectives

DCs play a pivotal role in priming naïve T cells and guiding their differentiation into effector and memory states. Different subsets of DCs induce distinct immune responses. cDC1s are critical for promoting CTL responses to tumors. However, because the TME is antagonistic to CD8^+^ T cells, the presence of cDC1s may impair anti-tumor immune responses, which occurs through their interference with the antigen uptake capacity of cDC2s and their responsiveness to migratory signals. In such contexts, cDC1s can act as an obstacle rather than a facilitator. Targeting cDC2s in these settings promotes DC migration, activates CD4^+^ T cells, and promotes anti-tumor immune responses. DCs can be engineered to ensure activation in the TME, recruiting more effector cells to the tumor side and facilitating continuous antigen sampling through direct killing by NK cells or CTLs or via the acquisition of newly released antigens due to immunogenic cell death, thereby contributing to de novo antigen-specific T cell immunity. This approach not only maintains tumor control through immunosurveillance but also protects CD8^+^ T cells from exhaustion in the tumor dLN. However, tumor heterogeneity poses challenges in cancer therapy [118].

The ICB therapies have achieved success in overcoming TME regulatory aspects, although their effectiveness is limited to specific patients and tumor types [119,120,121,122]. Understanding the mechanisms that enhance anti-tumor responses has the potential to improve the efficacy of ICBs by facilitating the development of new classes of immunotherapeutic agents [123]. Therefore, strategies to enhance DC functions offer new opportunities to improve ICB immunotherapy. The development of DC subtype-targeted therapies will contribute to the comprehensive design of combination immunotherapies. Further studies are necessary to elucidate DC heterogeneity and modify co-stimulatory molecules and metabolic cues, optimizing T cell activation in the dLN. This optimization should facilitate effective migration to the TME, recruiting anti-tumor effector T cells while preventing regulatory T cell activation. Implementing better strategies to optimize antigen processing and presentation and mitigate metabolic byproducts and overwhelming signaling from the TME could enhance immunotherapies for cancer treatment.
